# Phytosterol intake and overall survival in newly diagnosed ovarian cancer patients: An ambispective cohort study

**DOI:** 10.3389/fnut.2022.974367

**Published:** 2022-08-25

**Authors:** Jun-Qi Zhao, Ying-Ying Hao, Ting-Ting Gong, Yi-Fan Wei, Gang Zheng, Zong-Da Du, Bing-Jie Zou, Shi Yan, Fang-Hua Liu, Song Gao, Qi-Jun Wu, Yu-Hong Zhao

**Affiliations:** ^1^Department of Clinical Epidemiology, Shengjing Hospital of China Medical University, Shenyang, China; ^2^Clinical Research Center, Shengjing Hospital of China Medical University, Shenyang, China; ^3^Department of Obstetrics and Gynecology, Shengjing Hospital of China Medical University, Shenyang, China

**Keywords:** cohort, diet, ovarian cancer, phytosterol, survival

## Abstract

**Background:**

Phytosterol is a bioactive compound existing in all plant foods, which might have anticancer properties. The aim of this study was to first assess the impact of the pre-diagnosis phytosterol intake on overall survival (OS) of patients with ovarian cancer (OC).

**Materials and methods:**

This ambispective cohort study recruited 703 newly diagnosed OC patients to investigate the aforementioned associations. Dietary intake was assessed using a validated 111-item food frequency questionnaire. Deaths were ascertained until March 31, 2021, through active follow-up and medical records. Cox proportional hazards regression models were applied to calculate the hazard ratios (HRs) and 95% confidence intervals (CIs).

**Results:**

During the median follow-up of 37.17 months, 130 deaths occurred. The median age at diagnosis of 703 OC patients was 53.00 (interquartile: 48.00–60.00) years. Of these, almost half patients (48.08%) were diagnosed in advanced International Federation of Gynecology and Obstetrics (FIGO) stage (III-IV). Additionally, more than half patients were serous carcinoma (68.14%), poorly differentiated (85.21%), and no residual lesions (78.66%). Patients consumed the highest tertile of dietary campesterol (HR = 0.54, 95% CI = 0.31–0.94, *P* trend < 0.05), stigmasterol (HR = 0.60, 95% CI = 0.37–0.98), and β-sitosterol (HR = 0.63, 95% CI = 0.40–0.99) were significantly associated with better OS compared with those with the lowest tertile of intake. The curvilinear associations were observed between total phytosterols and β-sitosterol intake and OC survival (*P* non-linear < 0.05). Significant associations were generally consistent across different subgroups stratified by demographical, clinical, and immunohistochemical characteristics. Moreover, there were significant interactions between phytosterol intake and age at diagnosis, body mass index, as well as expressions of Wilms’ tumor-1 and Progestogen Receptor (all *P* interaction < 0.05).

**Conclusion:**

Pre-diagnosis higher campesterol, stigmasterol, and β-sitosterol intake were associated with better survival among OC patients.

## Introduction

Ovarian cancer (OC) is one of the most common malignant tumors in women, due to insidious onset and lack of typical symptoms, most patients were diagnosed in the advanced stages (1), and the mortality rate ranks first among gynecological malignancies (2). In 2020, there were 313,959 new cases of OC and 207,252 deaths worldwide (3). Despite surgical treatment and systematic treatment of OC have made great progress, the 5-year survival rate of developed regions still below 50% (4). Accumulated evidence indicated that several factors, including clinical stage (5), histological type (6), menopausal hormone therapy, and breastfeeding (7), were associated with the prognosis of OC. However, these factors are difficult to modify. Thus, it is particularly crucial to find modifiable prognostic factors that can improve the prognosis of OC. Of note, diet is a modifiable factor that could contribute to the improvement of OC survival, which has been verified by our previous research (8–10).

Emerging evidence has revealed that plant food consumption such as vegetables and fruits were inversely associated with OC survival (11). Phytosterol is a kind of bioactive compound found in all foods of plant origin (12), which is abundant in vegetables, fruits, legumes, cereals, and oils (13). Although more than 250 phytosterols have been reported so far, campesterol, β-sitosterol, stigmasterol, β-sitostanol, and campestanol are the main phytosterols found in food (14). Previous studies have suggested that phytosterol exhibited anticancer effect on multiple cancers (15–20). For example, McCann et al. found higher intake of stigmasterol was associated with the decreased risk of OC (20). Several epidemiological studies similarly indicated that phytosterol was related to the lower risk of stomach (15), colorectal (16), esophagus (17), breast (18), and lung (19) cancer. Potential biological mechanisms might account for the aforementioned associations, including promoting apoptosis, arresting the cell cycle (21), and reducing the production of reactive oxygen species (ROS) (22), all of which have been experimentally verified.

Notwithstanding, to our knowledge, no study has investigated the correlations between phytosterol intake and the survival among OC patients. Given the lack of prospective evidence, we performed the present study to evaluate the associations of pre-diagnosis phytosterol consumption with OC survival based on the Ovarian Cancer Follow-Up Study (OOPS).

## Materials and methods

### Study population

**FIGURE 1:**
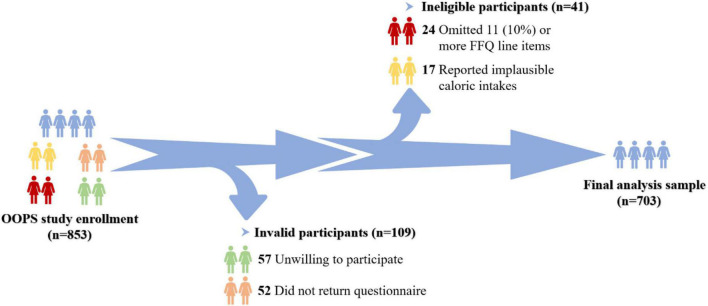
Flow of participants through the Ovarian Cancer Follow-Up Study (OOPS).

The OOPS is an ongoing ambispective cohort study of newly diagnosed OC patients, designed to assess the associations of demographic and clinical characteristics as well as lifestyle factors with the OC prognosis (8, 23, 24). During the time period between January 2015 and December 2020, 853 newly diagnosed OC patients aged 18–75 years were recruited. Among them, 796 women (93%) agreed to participate, and 744 (87%) women returned the completed study questionnaire. Furthermore, participants who omitted 11 (10%) or more food items (*n* = 24) and those reported implausible caloric intakes (< 500 or > 3,500 calories per day, *n* = 17) were excluded. Ultimately, aggregated to 703 women were eligible for the final analysis (Figure 1). The OOPS was approved by the Institutional Review Board of the Ethics Committee of Shengjing Hospital of China Medical University, Shenyang, China, and all women signed informed consent prior to participation.

### Data collection

Baseline information about socio-demographic and lifestyle characteristics, including education, income, smoking status (the smoking status before OC diagnosis), alcohol drinking (the alcohol drinking status before OC diagnosis), dietary change (defined as participants who had deliberately changed their eating habits recently with three response: 3 years ago, 1 to 2 years ago, and this year), menopausal status, parity, and physical activity, were recorded *via* self-administered questionnaires at the baseline interview. Anthropometrics were measured by trained personnel following a standard protocol, and body mass index (BMI) was calculated as weight in kilograms divided by height in squared meters. Physical activity was evaluated in OC patients according to previous study (25). In brief, all participants were required to report usual type and duration of activities related to work, commuting, household chores, and leisure-time exercise during the past 12 months (25). We calculated total physical activity through metabolic equivalent tasks (METs) from the 2011 update of a major compendium of physical activities (26). Clinical characteristics were extracted from the electronic medical records of the Shengjing hospital information system, including age at diagnosis, histological type, histopathologic grade, International Federation of Gynecology and Obstetrics (FIGO) stage, residual lesions, and comorbidities. On the basis of the routine protocol, specimens of OC tissues and adjacent tissues acquired after surgery were applied to immunohistochemistry (IHC) analysis. All indicators, including Wilms’ tumour-1 (WT-1), Estrogen Receptor (ER), Progesterone Receptor (PR), Vimentin, and p53, were divided into positive and negative. IHC expression was manually confirmed by two independent experienced pathologists.

### Dietary exposure and assessment

All participants were recruited at Shengjing Hospital of China Medical University, Shenyang, China and required to complete a 111-item semi-quantitative food frequency questionnaire (FFQ). In the present study, the FFQ was a modified version FFQ used in the Tianjin Chronic Low-grade Systemic Inflammation and Health cohort study (27). The FFQ was performed by well-trained and skilled researchers through face-to-face interviews. Pre-diagnosis dietary information was measured at baseline through the FFQ, its validity and reliability have been verified by previous studies (8, 9). The reproducibility coefficients (Spearman correlation coefficients and intraclass correlation coefficients) were above 0.5 for most food groups, and the correlation coefficients (Spearman correlation coefficients) were between 0.3 and 0.7 for most food groups between the FFQ and weighed dietary records (8–10). All newly diagnosed OC patients were required to report their usual intake frequency of each food item during the year prior to OC diagnosis, with seven response options: almost never, 2–3 times per month, 1 time per week, 2–3 times per week, 4–6 times per week, 1–2 times per day, and more than two times per day. The consumption of each food item was calculated by multiplying fitted portion sizes (gram/time) by the frequency at which each food item was consumed per day (28). Additionally, the Chinese food composition tables were applied as the nutrient database to obtain the nutrient content of each food item (29). By linking the information from the FFQ to the Chinese food composition table, the nutrient intake was calculated by first multiplying the amount of consumption for each food item by its nutrient content and subsequently summing nutrient contributions across all food items (30). The consumption of the following phytosterols from plant food were available for analysis: campestanol, β-sitostanol, campesterol, stigmasterol, and β-sitosterol. Besides, we calculated total phytosterols by summing the above variates. All nutrients were adjusted for total energy intake based on the residual method (31).

### Follow-up and outcome

At the present study, the outcome was overall survival (OS). All participants were followed up until the occurrence of mortality from any cause or the last follow-up (March 31, 2021). Information about the vital status of OC patients was ascertained from medical records every 6 months and by active follow-up. Survival time was calculated from the date of histologic OC diagnosis to the date of all-cause death or the date of the last follow-up for women who were still alive.

### Statistical analysis

Continuous variables were expressed as mean with standard deviation (SD) or median with interquartile (IQR). And categorical variables were expressed as number with percentage. Distribution of OC patients in terms of demographic and clinical characteristics across total phytosterols intake was examined using one-way ANOVA or Kruskal–Wallis test for continuous variables and Chi-square tests for categorical variables. We estimated crude OS probabilities and plotted crude survival curves by the Kaplan-Meier technique. The proportional hazards assumption was examined through adding an interaction term between each activity variable and log survival time, and variables in this analysis satisfied the conditions (all *P* > 0.05). Associations between pre-diagnosis phytosterol intake and OS of OC patients were examined using the Cox proportional hazards regression models. Phytosterol intake was categorized into tertiles, the hazard ratios (HRs) and 95% confidence intervals (CIs) were calculated using the lowest tertile served as the reference group. Continuous intakes of phytosterol were calculated by per SD increment. The linear trend test was assessed by assigning the median value of consumption for each tertile of phytosterol and treating it as a continuous variable in the respective regression model.

The final multivariate models were adjusted for age at diagnosis (< 50 or ≥ 50 years), education (junior secondary or below, senior high school/technical secondary school, and junior college/university or above), cigarette smoking (yes or no), alcohol drinking (yes or no), dietary change (yes or no), menopausal status (yes or no), parity (≤ 1 or ≥ 2), BMI (continuous, kg/m^2^), physical activity (continuous, MET/hours/day), FIGO stage (I–II, III–IV), histological type (serous or non-serous), histopathologic grade (well, moderately, and poorly differentiated), residual lesions (none, < 1, and ≥ 1 cm), comorbidities (yes or no), and total energy (continuous, kcal/day), isoflavone (continuous, mg/day), and monounsaturated fatty acid (continuous, g/day) intake. Selected covariates for the final models were based on correlation with phytosterol, clinical significance, and previous studies. Carbohydrate, total fiber, and polyunsaturated fatty acids intake excluded from the final analysis, due to the multicollinearity between covariates. We also conducted restricted cubic spline model with three knots (i.e., 5, 50, and 95th percentiles) to test the non-linear relationships between phytosterol intake and OC survival (32).

Stratified analyses were performed according to age at diagnosis (< 50 compared to ≥ 50 years), menopausal status (“no” compared to “yes”), BMI (< 25 compared to ≥ 25 kg/m^2^), histological type (serous compared to non-serous), FIGO stage (I-II compared to III-IV), and residual lesions (“no” compared to “yes”). Moreover, we also performed stratified analyses by IHC biomarkers, included WT-1 (“positive” compared to “negative”), ER (“positive” compared to “negative”), PR (“positive” compared to “negative”), Vimentin (“positive” compared to “negative”), and p53 (“positive” compared to “negative”). Potential interactions of phytosterol intake with these stratified variables were analyzed by adding cross-product terms in the multivariable Cox regression models. We implemented several sensitivity analyses to test the robustness of the primary findings. First, we adjusted the mean energy intake per day using the nutrient density method. Moreover, we excluded the patients having less than 1-year follow-up period to evaluate whether the associations were independent of follow-up periods. All statistical analyses were conducted by SAS version 9.4 (SAS Institute, Cary, NC, United States), and two-sided *P* values < 0.05 were considered statistically significant.

## Results

Socio-demographic and lifestyle characteristics of 703 OC patients were presented in Table 1, according to tertiles of total phytosterols intake. During the median follow-up 37.17 (IQR: 24.73–50.17) months, a total of 130 confirmed deaths were documented. The median age at diagnosis of 703 OC patients was 53.00 (IQR: 48.00–60.00) years. Of these, 48.65% were diagnosed in early FIGO stage (I-II), 48.08% were diagnosed in advanced FIGO stage (III-IV). In addition, among these OC patients, 68.14% were serous carcinoma, 13.94% were clear cell carcinoma, 12.52% were endometrioid carcinoma, and 2.28% were mucinous carcinoma. Moreover, most OC patients included in our study were poorly differentiated (85.21%) and no residual lesions (78.66%). OC patients with higher total phytosterols intake had longer follow-up time (*P* < 0.05). Moreover, participants with higher consumption of total phytosterols tended to consume more carbohydrate, total fiber, isoflavones, monounsaturated fatty acids, polyunsaturated fatty acids, and total energy (all *P* < 0.05). Advanced FIGO stage, larger residual lesions, and non-serous histological subtypes were statistically significant correlated to worse OC survival (Supplementary Table 1). Besides, we found the negative expressions of WT-1, ER, and PR were related to poorer survival of OC (Supplementary Table 2).

**TABLE 1:** Baseline characteristics of ovarian cancer patients by tertile of total phytosterols intake (*N* = 703).

Characteristics	Tertiles of total phytosterols intake (mg/d)*			*P*-value^†^
	T1	T2	T3	
Range	< 43.10	43.10–61.55	≥ 61.55	
No. of deaths/patients	51/234	34/234	45/235	0.12
Age at diagnosis (years), Median (IQR)	53.00 (48.00–61.00)	53.00 (47.00–60.00)	53.00 (48.00–60.00)	0.63
Follow-up time (months), Median (IQR)	27.35 (16.80–42.07)	31.99 (20.37–46.80)	35.43 (23.83–49.87)	< 0.05
Body mass index (kg/m^2^), Median (IQR)	23.30 (21.00–25.10)	23.15 (20.60–25.20)	23.10 (20.80–24.90)	0.72
Physical activity (MET/hours/day), Median (IQR)	14.40 (7.00–22.30)	13.65 (6.30–22.00)	14.10 (6.10–22.40)	0.90
Ever cigarette smoking	27 (11.54)	17 (7.26)	24 (10.21)	0.28
Ever alcohol drinking	58 (24.79)	50 (21.37)	41 (17.45)	0.15
Ever dietary change	59 (25.21)	51 (21.79)	58 (24.68)	0.65
Ever menopause	167 (71.37)	164 (70.09)	177 (75.32)	0.42
Parity				0.10
≤ 1	179 (76.50)	167 (71.37)	159 (67.66)	
≥ 2	55 (23.50)	67 (28.63)	76 (32.34)	
Educational level				0.62
Junior secondary or below	118 (50.43)	127 (54.27)	130 (55.32)	
Senior high school/technical secondary school	57 (24.36)	46 (19.66)	44 (18.72)	
Junior college/university or above	59 (25.21)	61 (26.07)	61 (25.96)	
Income per month (Yuan)				0.39
< 5,000	138 (58.97)	150 (64.10)	133 (56.60)	
5,000 to < 10,000	70 (29.91)	55 (23.50)	69 (29.36)	
≥ 10,000	26 (11.12)	29 (12.40)	33 (14.04)	
Mean (SD) total energy intake (kcal/d)	1573.36 (581.90)	1279.23 (476.07)	1514.41 (552.23)	< 0.05
Mean (SD) carbohydrate intake (g/d)*	232.67 (25.53)	225.19 (22.29)	222.85 (27.81)	< 0.05
Mean (SD) total fiber intake (g/d)*	13.20 (3.34)	17.05 (2.47)	22.27 (4.49)	< 0.05
Mean (SD) isoflavone intake (mg/d)*	7.98 (7.89)	15.82 (9.22)	26.35 (15.93)	< 0.05
Mean (SD) monounsaturated fatty acid intake (g/d)*	8.85 (3.47)	9.55 (2.57)	9.45 (3.62)	< 0.05
Mean (SD) polyunsaturated fatty acid intake (g/d)*	4.12 (1.59)	5.31 (1.49)	6.45 (2.53)	< 0.05

IQR, interquartile; MET, metabolic equivalents of task; SD, standard deviation; T, tertile.

*Adjusted for energy by the residual method.

^†^*P*-values were determined with one-way ANOVA or Kruskal–Wallis test for continuous variables, and the chi-square test for categorical variables.

Values are numbers (percentages) unless stated otherwise.

**FIGURE 2:**
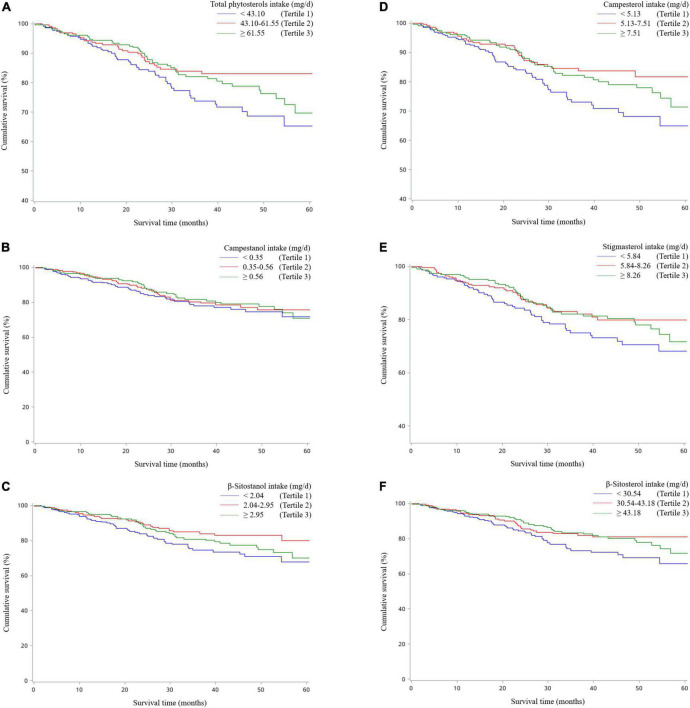
Kaplan–Meier survival curves for total phytosterols **(A)**, campestanol **(B)**, β-sitostanol **(C)**, campesterol **(D)**, stigmasterol **(E)**, and β-sitosterol **(F)**.

**FIGURE 3:**
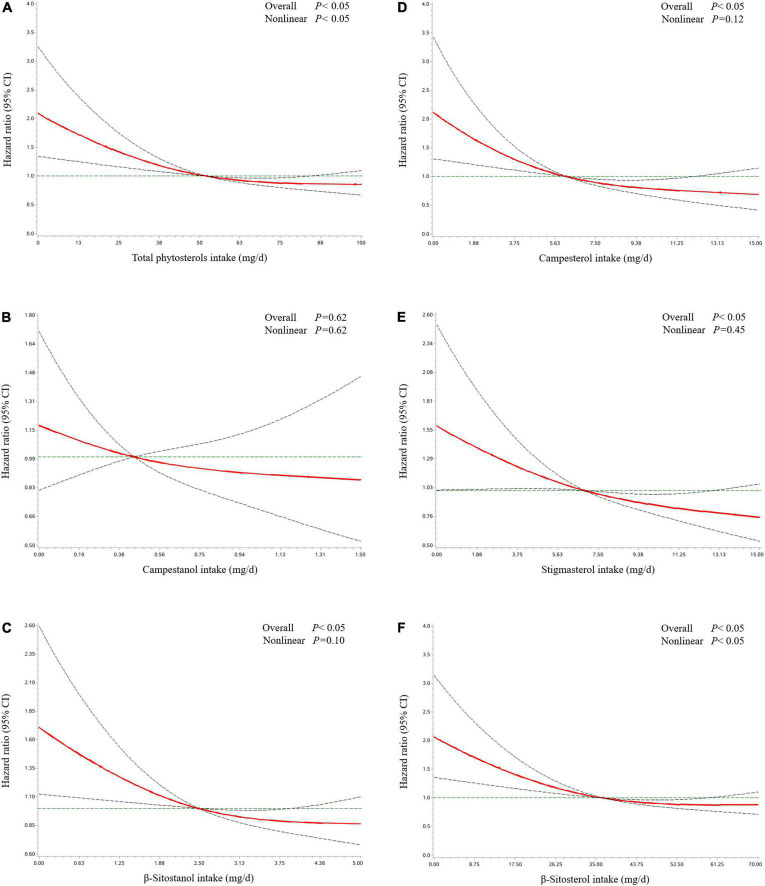
Hazard ratio and 95% confidence interval of overall survival among ovarian cancer patients by total phytosterols **(A)**, campestanol **(B)**, β-sitostanol **(C)**, campesterol **(D)**, stigmasterol **(E)**, and β-sitosterol **(F)**. The associations were adjusted for age at diagnosis, education, cigarette smoking, alcohol drinking, monthly household income, dietary change, menopausal status, parity, body mass index, physical activity, federation of gynecology and obstetrics stage, histological type, histopathologic grade, residual lesions, comorbidities, and total energy, isoflavone, and monounsaturated fatty acid intake. The red line and dashed line represent the estimated HRs and their 95% CIs, respectively. CI, confidence interval; HR, hazard ratio; OC, ovarian cancer.

Table 2 revealed the associations of dietary phytosterol intake with OS of OC patients. After controlling for potential confounders, although we failed to observe significant association between total phytosterols and OC survival (HR_*T3 vs. T1*_ = 0.64, 95% CI = 0.40–1.03), we found that the highest tertile of campesterol intake was associated with better OS than the lowest tertile of intake (HR = 0.54, 95% CI = 0.31–0.94) with an evident linear trend (*P* trend < 0.05) (Figure 2). Moreover, dietary β-sitosterol intake was related to the favorable OC survival (HR_*T3 vs. T1*_ = 0.63, 95% CI = 0.40–0.99), similar pattern was also noticed in stigmasterol (HR_*T3 vs. T1*_ = 0.60, 95% CI = 0.37–0.98) (Figure 2). However, we failed to observe significant linear trend in these aforementioned variables. Furthermore, our results suggested that there probably existed curvilinear relationships between total phytosterols and β-sitosterol intake and OC survival (*P* non-linear < 0.05) (Figure 3).

**TABLE 2:** Adjusted hazard ratio (HR) and 95% confidence interval (CI) for the associations of phytosterol intake with overall survival among 703 ovarian cancer patients*.

Characteristics	Tertiles of intake (mg/d)**			*P* trend^†^	Continuous^‡^
**Total phytosterols**	< 43.10	43.10–61.55	≥ 61.55		
Deaths, N (% of total deaths)	51 (39.23)	34 (26.15)	45 (34.62)		
Model 1	1.00 (Ref)	0.59 (0.38–0.92)	0.73 (0.49–1.09)	0.18	0.84 (0.70–1.00)
Model 2	1.00 (Ref)	0.56 (0.36–0.88)	0.71 (0.47–1.06)	0.14	0.83 (0.69–1.00)
Model 3	1.00 (Ref)	0.49 (0.31–0.79)	0.64 (0.40–1.03)	0.11	0.76 (0.59–0.97)
**Campestanol**	< 0.35	0.35–0.56	≥ 0.56		
Deaths, N (% of total deaths)	46 (35.38)	42 (32.31)	42 (32.31)		
Model 1	1.00 (Ref)	0.90 (0.59–1.38)	0.86 (0.56–1.30)	0.49	0.90 (0.76–1.08)
Model 2	1.00 (Ref)	0.78 (0.51–1.20)	0.80 (0.52–1.23)	0.38	0.89 (0.73–1.07)
Model 3	1.00 (Ref)	0.73 (0.46–1.16)	0.70 (0.38–1.30)	0.31	0.77 (0.51–1.16)
**β -Sitostanol**	< 2.04	2.04–2.95	≥ 2.95		
Deaths, N (% of total deaths)	52 (40.00)	32 (24.62)	46 (35.38)		
Model 1	1.00 (Ref)	0.58 (0.37–0.91)	0.77 (0.52–1.15)	0.30	0.87 (0.72–1.04)
Model 2	1.00 (Ref)	0.55 (0.35–0.86)	0.77 (0.52–1.15)	0.32	0.86 (0.71–1.03)
Model 3	1.00 (Ref)	0.48 (0.30–0.76)	0.65 (0.39–1.09)	0.16	0.72 (0.54–0.96)
**Campesterol**	< 5.13	5.13–7.51	≥ 7.51		
Deaths, N (% of total deaths)	53 (40.77)	33 (25.38)	44 (33.85)		
Model 1	1.00 (Ref)	0.55 (0.36–0.87)	0.69 (0.46–1.03)	0.11	0.85 (0.71–1.01)
Model 2	1.00 (Ref)	0.51 (0.33–0.81)	0.64 (0.43–0.97)	0.06	0.84 (0.70–1.00)
Model 3	1.00 (Ref)	0.52 (0.33–0.83)	0.54 (0.31–0.94)	< 0.05	0.67 (0.47–0.94)
**Stigmasterol**	< 5.84	5.84–8.26	≥ 8.26		
Deaths, N (% of total deaths)	52 (40.00)	36 (27.69)	42 (32.31)		
Model 1	1.00 (Ref)	0.68 (0.44–1.05)	0.72 (0.48–1.08)	0.14	0.81 (0.68–0.97)
Model 2	1.00 (Ref)	0.60 (0.39–0.94)	0.67 (0.44–1.02)	0.09	0.79 (0.66–0.96)
Model 3	1.00 (Ref)	0.53 (0.33–0.84)	0.60 (0.37–0.98)	0.07	0.69 (0.53–0.90)
**ß-Sitosterol**	< 30.54	30.54–43.18	≥ 43.18		
Deaths, N (% of total deaths)	51 (39.23)	36 (27.69)	43 (33.08)		
Model 1	1.00 (Ref)	0.66 (0.43–1.02)	0.67 (0.45–1.01)	0.07	0.84 (0.70–1.01)
Model 2	1.00 (Ref)	0.66 (0.43–1.02)	0.67 (0.44–1.01)	0.07	0.83 (0.69–1.00)
Model 3	1.00 (Ref)	0.61 (0.39–0.97)	0.63 (0.40–0.99)	0.06	0.79 (0.63–0.99)

BMI, body mass index; CI, confidence interval; HR, hazard ratio; Ref, reference.

*Hazard ratio (HR) and 95% CI were calculated through the Cox proportional hazards regression model.

**Adjusted for energy by the residual method.

^†^Test for trend based on variables containing the median value for each group.

^‡^Continuous intakes were calculated by per standard deviation increase.

Model 1 was adjusted for age at diagnosis (< 50 or ≥ 50 years) and total energy (continuous, kcal/day).

Model 2 same as Model 1 and further adjusted for education (junior secondary or below, senior high school/technical secondary school, and junior college/university or above), cigarette smoking (yes or no), alcohol drinking (yes or no), monthly household income (< 5, 5–10, ≥ 10 RMB; thousand yuan), dietary change (yes or no), menopausal status (yes or no), parity (≤ 1, ≥ 2), body mass index (continuous, kg/m^2^), and physical activity (continuous, MET/hours/day).

Model 3 same as Model 2 and further adjusted for International Federation of Gynecology and Obstetrics (FIGO) stage (I–II, III–IV, and unknown), histological type (serous or non-serous), histopathologic grade (well, moderate, and poorly differentiated), residual lesions (none, < 1, and ≥ 1 cm), and comorbidities (yes or no), isoflavone (continuous, mg/day), and monounsaturated fatty acid intake (continuous, g/day).

Consistent findings from subgroup analyses were still observed that higher phytosterol intake was related to better OS of OC patients with I-II FIGO stage, BMI ≥ 25, residual lesions, age at diagnosis > 50, or postmenopausal patients. Additionally, the results of subgroup analyses stratified by IHC biomarkers showed that inversely significant associations were presented in patients with the negative expressions of WT-1, ER, and PR as well as the positive expressions of Vimentin and p53 (Tables 3, 4). Notably, we found significant interactions of age at diagnosis, BMI, and the expressions of WT-1 and PR on the correlations between phytosterol intake and OS of OC patients (all *P* interaction < 0.05).

**TABLE 3:** Subgroup analyses of demographical, clinical, and immunohistochemical characteristics for adjusted hazard ratio (HR) and 95% confidence interval (CI) for the associations of total phytosterols, campestanol, and β-sitostanol intake with overall survival among ovarian cancer patients.

Characteristics	Tertiles of intake*											
	Total phytosterols				Campestanol				β -Sitostanol			
	T1	T2	T3	*P*_interaction_ **	T1	T2	T3	*P*_interaction_ **	T1	T2	T3	*P*_interaction_ **
**Age at diagnosis (years)**				0.17				0.20				0.09
≤ 50	1.00 (Ref)	0.30 (0.12–0.78)	0.64 (0.25–1.60)		1.00 (Ref)	0.43 (0.17–1.10)	0.70 (0.21–2.37)		1.00 (Ref)	0.63 (0.27–1.48)	0.87 (0.31–2.43)	
> 50	1.00 (Ref)	0.50 (0.27–0.90)	0.61 (0.34–1.12)		1.00 (Ref)	0.85 (0.48–1.50)	0.66 (0.31–1.41)		1.00 (Ref)	0.49 (0.27–0.87)	0.58 (0.31–1.09)	
**Menopausal status**				0.42				0.92				0.40
No	1.00 (Ref)	0.45 (0.14–1.47)	0.63 (0.21–1.90)		1.00 (Ref)	0.37 (0.12–1.10)	0.95 (0.23–3.85)		1.00 (Ref)	0.38 (0.13–1.12)	0.53 (0.16–1.76)	
Yes	1.00 (Ref)	0.47 (0.26–0.83)	0.57 (0.32–1.01)		1.00 (Ref)	0.96 (0.55–1.68)	0.83 (0.39–1.74)		1.00 (Ref)	0.50 (0.29–0.86)	0.51 (0.28–0.95)	
**Body mass index (kg/m^2^)**				0.06				0.25				< 0.05
<25	1.00 (Ref)	0.47 (0.27–0.83)	0.64 (0.37–1.10)		1.00 (Ref)	0.99 (0.57–1.72)	1.27 (0.62–2.59)		1.00 (Ref)	0.51 (0.29–0.87)	0.80 (0.45–1.42)	
≥ 25	1.00 (Ref)	0.38 (0.12–1.21)	0.58 (0.19–1.82)		1.00 (Ref)	0.30 (0.11–0.84)	0.09 (0.02–0.36)		1.00 (Ref)	0.41 (0.13–1.30)	0.22 (0.06–0.78)	
**Histological type**				0.18				0.06				0.11
Serous	1.00 (Ref)	0.54 (0.30–0.97)	0.65 (0.37–1.16)		1.00 (Ref)	0.84 (0.48–1.46)	0.88 (0.42–1.85)		1.00 (Ref)	0.49 (0.28–0.86)	0.59 (0.32–1.09)	
Non-serous	1.00 (Ref)	0.48 (0.18–1.25)	0.90 (0.34–2.39)		1.00 (Ref)	0.46 (0.18–1.18)	0.66 (0.18–2.43)		1.00 (Ref)	0.41 (0.15–1.09)	1.44 (0.49–4.23)	
**FIGO stage**				0.76				0.78				0.52
I-II	1.00 (Ref)	0.26 (0.10–0.65)	0.36 (0.14–0.95)		1.00 (Ref)	0.77 (0.34–1.73)	0.50 (0.15–1.65)		1.00 (Ref)	0.44 (0.19–1.02)	0.61 (0.22–1.67)	
III-IV	1.00 (Ref)	0.70 (0.39–1.26)	0.85 (0.47–1.55)		1.00 (Ref)	0.72 (0.39–1.31)	0.94 (0.43–2.04)		1.00 (Ref)	0.55 (0.31–0.98)	0.76 (0.41–1.40)	
**Residual lesions**				0.81				0.32				0.59
No	1.00 (Ref)	0.58 (0.32–1.04)	0.69 (0.37–1.29)		1.00 (Ref)	0.65 (0.36–1.16)	0.67 (0.31–1.43)		1.00 (Ref)	0.59 (0.33–1.06)	0.78 (0.39–1.55)	
Yes	1.00 (Ref)	0.36 (0.15–0.85)	0.53 (0.24–1.17)		1.00 (Ref)	1.14 (0.50–2.59)	0.93 (0.28–3.08)		1.00 (Ref)	0.44 (0.20–0.97)	0.44 (0.19–1.01)	
**WT-1**				< 0.05				< 0.05				< 0.05
Positive	1.00 (Ref)	0.46 (0.21–1.01)	0.64 (0.31–1.34)		1.00 (Ref)	1.29 (0.63–2.63)	0.73 (0.27–1.96)		1.00 (Ref)	0.37 (0.17–0.81)	0.85 (0.39–1.84)	
Negative	1.00 (Ref)	0.43 (0.18–1.03)	0.23 (0.08–0.72)		1.00 (Ref)	0.45 (0.18–1.12)	0.54 (0.14–2.13)		1.00 (Ref)	0.49 (0.21–1.16)	0.30 (0.09–0.99)	
**ER**				0.11				0.46				0.26
Positive	1.00 (Ref)	0.58 (0.31–1.09)	0.78 (0.43–1.42)		1.00 (Ref)	0.84 (0.47–1.50)	0.54 (0.24–1.21)		1.00 (Ref)	0.57 (0.31–1.03)	0.74 (0.39–1.42)	
Negative	1.00 (Ref)	0.74 (0.25–2.21)	0.52 (0.11–2.49)		1.00 (Ref)	0.30 (0.09–1.06)	0.23 (0.04–1.40)		1.00 (Ref)	0.29 (0.08–1.04)	0.44 (0.09–2.24)	
**PR**				< 0.05				0.31				0.13
Positive	1.00 (Ref)	0.91 (0.41–2.06)	0.96 (0.43–2.15)		1.00 (Ref)	1.32 (0.60–2.89)	0.88 (0.27–2.82)		1.00 (Ref)	1.13 (0.50–2.53)	1.29 (0.55–3.03)	
Negative	1.00 (Ref)	0.37 (0.18–0.76)	0.52 (0.25–1.11)		1.00 (Ref)	0.50 (0.25–1.02)	0.34 (0.13–0.91)		1.00 (Ref)	0.22 (0.10–0.47)	0.37 (0.16–0.85)	
**Vimentin**				0.92				0.77				0.73
Positive	1.00 (Ref)	0.35 (0.11–1.07)	0.42 (0.13–1.38)		1.00 (Ref)	0.96 (0.28–3.27)	0.49 (0.08–2.93)		1.00 (Ref)	0.49 (0.16–1.51)	0.38 (0.10–1.47)	
Negative	1.00 (Ref)	0.52 (0.26–1.07)	0.72 (0.36–1.47)		1.00 (Ref)	0.79 (0.40–1.53)	0.53 (0.21–1.34)		1.00 (Ref)	0.41 (0.20–0.84)	0.54 (0.25–1.16)	
**p53**				0.55				0.78				0.63
Positive	1.00 (Ref)	0.40 (0.22–0.70)	0.57 (0.31–1.03)		1.00 (Ref)	0.82 (0.48–1.42)	0.56 (0.25–1.23)		1.00 (Ref)	0.44 (0.25–0.78)	0.71 (0.38–1.34)	
Negative	1.00 (Ref)	0.42 (0.12–1.44)	0.74 (0.23–2.42)		1.00 (Ref)	1.02 (0.33–3.20)	0.73 (0.18–3.03)		1.00 (Ref)	0.37 (0.10–1.30)	0.50 (0.13–1.91)	

CI, confidence interval; ER, Estrogen Receptor; FIGO, International Federation of Gynecology and Obstetrics; HR, hazard ratio; PR, Progestogen Receptor; Ref, reference; T, tertile; WT-1, Wilms’ tumor-1.

*Adjusted for energy by the residual method.

**Test for interaction based on strata and phytosterol intake.

Hazard ratio and 95% CI were calculated with the use of the Cox proportional hazards regression model with adjustment for age at diagnosis, education, cigarette smoking, alcohol drinking, monthly household income, dietary change, menopausal status, parity, body mass index, physical activity, FIGO stage, histological type, histopathologic grade, residual lesions, comorbidities, and total energy, isoflavone, and monounsaturated fatty acid intake.

**TABLE 4:** Subgroup analyses of demographical, clinical, and immunohistochemical characteristics for adjusted hazard ratio (HR) and 95% confidence interval (CI) for the associations of campesterol, stigmasterol, and β-sitosterol intake with overall survival among ovarian cancer patients.

Characteristics	Tertiles of intake*											
	Campesterol				Stigmasterol				β -Sitosterol			
	T1	T2	T3	*P*_interaction_ **	T1	T2	T3	*P*_interaction_ **	T1	T2	T3	*P*_interaction_ **
**Age at diagnosis (years)**				0.24				< 0.05				0.34
≤ 50	1.00 (Ref)	0.51 (0.22–1.22)	0.50 (0.16–1.58)		1.00 (Ref)	0.68 (0.28–1.63)	0.95 (0.37–2.42)		1.00 (Ref)	0.51 (0.22–1.22)	0.68 (0.29–1.60)	
> 50	1.00 (Ref)	0.48 (0.26–0.86)	0.53 (0.27–1.03)		1.00 (Ref)	0.52 (0.29–0.92)	0.47 (0.25–0.90)		1.00 (Ref)	0.60 (0.33–1.08)	0.59 (0.33–1.07)	
**Menopausal status**				0.65				0.15				0.65
No	1.00 (Ref)	0.52 (0.18–1.52)	0.63 (0.19–2.09)		1.00 (Ref)	1.26 (0.45–3.55)	0.75 (0.26–2.22)		1.00 (Ref)	0.58 (0.20–1.72)	0.81 (0.30–2.20)	
Yes	1.00 (Ref)	0.45 (0.25–0.80)	0.53 (0.28–1.03)		1.00 (Ref)	0.47 (0.27–0.83)	0.49 (0.27–0.90)		1.00 (Ref)	0.53 (0.31–0.93)	0.56 (0.32–0.96)	
**Body mass index (kg/m^2^)**				0.15				< 0.05				0.20
< 25	1.00 (Ref)	0.48 (0.28–0.83)	0.56 (0.31–1.04)		1.00 (Ref)	0.52 (0.30–0.89)	0.64 (0.36–1.14)		1.00 (Ref)	0.64 (0.37–1.09)	0.60 (0.35–1.01)	
≥ 25	1.00 (Ref)	1.42 (0.47–4.31)	0.69 (0.15–3.21)		1.00 (Ref)	0.66 (0.21–2.01)	0.40 (0.12–1.26)		1.00 (Ref)	0.45 (0.16–1.27)	0.80 (0.29–2.24)	
**Histological type**				0.09				0.06				0.33
Serous	1.00 (Ref)	0.54 (0.31–0.94)	0.59 (0.31–1.13)		1.00 (Ref)	0.51 (0.29–0.90)	0.63 (0.35–1.12)		1.00 (Ref)	0.66 (0.38–1.16)	0.70 (0.41–1.21)	
Non-serous	1.00 (Ref)	0.45 (0.18–1.13)	1.10 (0.31–3.87)		1.00 (Ref)	0.55 (0.23–1.32)	0.66 (0.22–1.93)		1.00 (Ref)	0.41 (0.16–1.01)	0.65 (0.25–1.68)	
**FIGO stage**				0.87				0.43				0.93
I-II	1.00 (Ref)	0.21 (0.08–0.52)	0.24 (0.08–0.75)		1.00 (Ref)	0.30 (0.12–0.73)	0.47 (0.17–1.32)		1.00 (Ref)	0.37 (0.16–0.86)	0.33 (0.13–0.82)	
III-IV	1.00 (Ref)	0.78 (0.44–1.38)	0.84 (0.42–1.68)		1.00 (Ref)	0.71 (0.40–1.25)	0.75 (0.41–1.39)		1.00 (Ref)	1.02 (0.58–1.81)	0.88 (0.49–1.57)	
**Residual lesions**				0.94				0.95				0.85
No	1.00 (Ref)	0.53 (0.29–0.95)	0.63 (0.31–1.28)		1.00 (Ref)	0.50 (0.27–0.90)	0.59 (0.30–1.15)		1.00 (Ref)	0.70 (0.39–1.25)	0.62 (0.33–1.14)	
Yes	1.00 (Ref)	0.35 (0.16–0.79)	0.22 (0.08–0.67)		1.00 (Ref)	0.56 (0.25–1.26)	0.71 (0.30–1.67)		1.00 (Ref)	0.54 (0.24–1.21)	0.46 (0.22–0.98)	
**WT-1**				< 0.05				< 0.05				< 0.05
Positive	1.00 (Ref)	0.48 (0.23–1.01)	0.42 (0.18–1.01)		1.00 (Ref)	0.70 (0.33–1.48)	0.89 (0.41–1.91)		1.00 (Ref)	0.75 (0.36–1.56)	0.68 (0.33–1.41)	
Negative	1.00 (Ref)	0.35 (0.14–0.87)	0.38 (0.13–1.15)		1.00 (Ref)	0.41 (0.17–0.98)	0.19 (0.06–0.59)		1.00 (Ref)	0.47 (0.21–1.07)	0.17 (0.06–0.50)	
**ER**				0.20				0.17				0.15
Positive	1.00 (Ref)	0.56 (0.30–1.02)	0.57 (0.28–1.16)		1.00 (Ref)	0.49 (0.26–0.91)	0.88 (0.48–1.64)		1.00 (Ref)	0.80 (0.43–1.49)	0.82 (0.46–1.48)	
Negative	1.00 (Ref)	0.28 (0.09–0.91)	0.30 (0.05–1.71)		1.00 (Ref)	0.38 (0.11–1.29)	0.12 (0.02–0.74)		1.00 (Ref)	0.61 (0.20–1.80)	0.56 (0.14–2.28)	
**PR**				< 0.05				0.05				< 0.05
Positive	1.00 (Ref)	0.62 (0.27–1.40)	0.60 (0.24–1.53)		1.00 (Ref)	0.56 (0.25–1.27)	0.97 (0.44–2.16)		1.00 (Ref)	1.06 (0.47–2.37)	0.97 (0.45–2.12)	
Negative	1.00 (Ref)	0.44 (0.22–0.87)	0.49 (0.20–1.19)		1.00 (Ref)	0.38 (0.19–0.79)	0.37 (0.17–0.82)		1.00 (Ref)	0.49 (0.23–1.03)	0.51 (0.25–1.05)	
**Vimentin**				0.84				0.98				0.81
Positive	1.00 (Ref)	0.10 (0.02–0.43)	0.39 (0.10–1.50)		1.00 (Ref)	0.28 (0.09–0.90)	0.35 (0.11–1.15)		1.00 (Ref)	0.15 (0.04–0.58)	0.27 (0.08–0.97)	
Negative	1.00 (Ref)	0.56 (0.28–1.09)	0.50 (0.21–1.15)		1.00 (Ref)	0.48 (0.23–0.98)	0.91 (0.44–1.88)		1.00 (Ref)	0.73 (0.37–1.46)	0.81 (0.42–1.58)	
**p53**				0.68				0.42				0.66
Positive	1.00 (Ref)	0.51 (0.30–0.89)	0.44 (0.22–0.91)		1.00 (Ref)	0.68 (0.39–1.18)	0.57 (0.30–1.08)		1.00 (Ref)	0.46 (0.26–0.81)	0.51 (0.29–0.91)	
Negative	1.00 (Ref)	0.36 (0.10–1.23)	0.57 (0.13–2.41)		1.00 (Ref)	0.18 (0.05–0.73)	0.50 (0.13–1.96)		1.00 (Ref)	0.50 (0.15–1.65)	0.63 (0.20–2.00)	

CI, confidence interval; ER, Estrogen Receptor; FIGO, International Federation of Gynecology and Obstetrics; HR, hazard ratio; PR, Progestogen Receptor; Ref, reference; T, tertile; WT-1, Wilms’ tumor-1.

*Adjusted for energy by the residual method.

**Test for interaction based on strata and phytosterol intake.

Hazard ratio and 95% CI were calculated with the use of the Cox proportional hazards regression model with adjustment for age at diagnosis, education, cigarette smoking, alcohol drinking, monthly household income, dietary change, menopausal status, parity, body mass index, physical activity, FIGO stage, histological type, histopathologic grade, residual lesions, comorbidities, and total energy, isoflavone, and monounsaturated fatty acid intake.

In sensitivity analyses that adjusted energy with nutrient density method, we found the association between campesterol intake and the OS of OC patients remained significant (Supplementary Table 3). However, we failed to find statistically significant correlations between β-sitosterol and stigmasterol intake with OC survival. Additionally, after excluding the patients less than 1-year follow-up, we found OC patients consumed the highest tertile of dietary total phytosterols, campesterol, and β-sitosterol were significantly associated with better OS compared with those with the lowest tertile of intake (data not shown).

## Discussion

In this ambispective cohort study, we firstly examined the correlations between pre-diagnosis phytosterol intake and OS of OC patients. We found that higher campesterol, stigmasterol, and β-sitosterol intake were associated with better OS, total phytosterols and other phytosterols showed null association. Interestingly, we observed significant interactions of age at diagnosis, BMI, and the expressions of WT-1 and PR on the associations of phytosterol intake with OC survival.

Although no research has investigated the relationships between dietary phytosterol consumption and OC survival, one study examined the associations of phytosterol intake with the risk of OC (20). McCann et al. performed a case-control study in western New York involving 124 OC cases and 696 population-based controls, found higher stigmasterol intake was associated with a decreased risk of OC (OR = 0.42, 95% CI = 0.20–0.87) (20). Moreover, several previous studies have provided potential significant evidence for other cancer outcomes (15–19). For example, a hospital-based case-control study conducted in Guangzhou, China, enrolled 1,802 colorectal cases and 1,813 controls, suggested that dietary consumptions of total phytosterols, β-sitosterol, campesterol, and campestanol were inversely associated with colorectal cancer risk, whereas stigmasterol intake was related to an increased risk of colorectal cancer (16). Additionally, several case-control studies implemented in Uruguay with 100–500 newly diagnosed multiple cancer patients manifested that phytosterol were related to the decreased risk of stomach cancer (15), esophageal cancer (17), breast cancer (18), and lung cancer (19). Generally, current studies suggested that phytosterol might have favorable effect on cancers. Given the lack of relevant literature on phytosterol intake and OC survival, further studies are warranted to validate our results.

Despite null association was observed between β-sitostanol intake and OC survival, findings from stratified analysis by BMI showed that β-sitostanol intake was associated with better OS of OC patients among the subgroups of high BMI (BMI ≥ 25 kg/m^2^). Of note, we also observed a significant interaction between β-sitostanol intake and BMI (*P* interaction < 0.05). Since one previous meta-analysis reported that high BMI at diagnosis was not correlated to the prognosis of OC (33). Moreover, the distribution of death in present study were mainly concentrated in the normal BMI range. Hence, the inverse association presented in high BMI patients might be attributed to the interaction between BMI and β-sitostanol consumption. Our findings need to be validated by future studies.

Vitro experiments have shown that several phytosterols, including campesterol, β-sitosterol, and stigmasterol, exerted anticancer effect on OC, which provided potential biological mechanisms for our findings. These phytosterols activated proapoptotic signals and induced the apoptosis of OC cells, simultaneously upregulated the calcium levels of cytosolic and mitochondrial and caused excessive calcium overload (34–36), which further promoted the cell apoptosis (37, 38). Besides, these phytosterols promoted the overexpression of unfolded protein response and endoplasmic reticulum-mitochondria axis signals, which triggered cell apoptosis pathway and autophagic stimulus and caused endoplasmic reticulum stress (34–36, 39), the accumulation of endoplasmic reticulum stress induced the cancer cells death (40). Moreover, these phytosterols promoted the production of ROS (34–36), excessive ROS caused cell death through intrinsic apoptotic signals in the mitochondria or extrinsic apoptotic signals by death receptor pathways (35, 41, 42). Additionally, these phytosterols inhibited cell growth and cell cycle progression through inhibiting the expression of the proliferating cell nuclear antigen and PI3K/MAPK signal pathways, similarly reduced OC cells migration and inhibited the aggregation of OC cells (34–36). Despite present evidence suggested phytosterol could improve OC survival, the vitro trials only conducted in the ES2 and OV90 human OC cells and restricted to several phytosterols. Future research should further explore the effect of other phytosterols and total phytosterols on multiple types of OC cells and conduct randomized controlled trial to illustrate exact and detailed biological mechanisms.

Previous studies suggested that the expression of IHC biomarkers, including ER, PR, p53, WT-1, and Vimentin, might have prognostic implications on the OC survival (43–45). Notably, our study found significant interactions between pre-diagnosis phytosterol intake and PR as well as WT-1 in relation to the survival of OC patients. Nevertheless, restricted to the fewer participants in some categories, we could not fully eliminate the possibility of accidental findings. Future studies are needed to validate these interactions.

A major strength of the present study is that we firstly investigated pre-diagnosis phytosterol intake and OS of OC patients. Second, the OOPS was an ambispective cohort study with high participation rates (93%) and a small proportion of loss to follow-up (6%), which could reduce the likelihood of recall bias and selection bias. Third, the comprehensive collection of detailed lifestyle and clinical characteristics in relation to OC survival allowed us to rigorously control potential confounding factors and provide more credible findings. Moreover, we performed multifaceted subgroup analyses and considered the interactions of phytosterol with several key influential factors to strength the reliability of primary results.

Nonetheless, the present study also has some potential limitations. First, the baseline data, including dietary intake, was collected through a self-administered questionnaire, which might lead to recall bias. However, we used a highly reproducible validated FFQ, which was performed by well-trained and skilled researchers through face-to-face interviews. Second, our study only collected dietary intake prior to diagnosis, some participants might change their dietary habits anterior to the year before diagnosis. However, only a small proportion of participants (23.9%) of our study changed their dietary habits and we controlled for dietary change in the final analysis. Third, we failed to evaluate the associations of dietary phytosterol intake with OC specific mortality, due to the data for the cause of death were not obtainable. However, previous study indicated that the results of overall and OC-specific mortality were highly consistent (46). Fourth, we did not obtain the consumption of cooking oil, the consumption of total phytosterols in current study (mean: 55.1 mg/d) was obviously lower than the consumption of total phytosterols in United States (mean for cases: 557.8 mg/d; mean for controls: 646.8 mg/d); thus, it should be cautious to generalize our findings to western populations. Fifth, although we have collected the information about surgery and chemotherapy, the details of these information are relatively limited, potential residual confounding of different surgical and chemotherapy protocols on the associations between phytosterol intake and OC survival might not have been ruled out completely. Sixth, since the present study is a single-center cohort study, several biases such as selection bias are unavoidable. Last, although many confounders were considered, the impact of unknown or unmeasured factors might not be eliminated in any of the observational studies.

## Conclusion

In conclusion, the present study provides evidence suggesting that the pre-diagnosis higher consumption of campesterol, β-sitosterol, and stigmasterol are associated with better OS of OC patients. Further studies with extended follow-up period and larger sample size are warranted to confirm our findings.

## Data availability statement

The original contributions presented in this study are included in the article/Supplementary material, further inquiries can be directed to the corresponding authors.

## Ethics statement

The studies involving human participants were reviewed and approved by Institutional Review Board of the Ethics Committee of Shengjing Hospital of China Medical University, Shenyang, China. The patients/participants provided their written informed consent to participate in this study.

## Author contributions

J-QZ, Y-YH, T-TG, Q-JW, and Y-HZ conceived the study. T-TG, SG, Q-JW, and Y-HZ contributed to the design. T-TG, SY, and SG collected the data. Y-FW, SY, F-HL, and Q-JW cleaned the data and checked the discrepancy. J-QZ, Y-FW, and F-HL analyzed the data. All authors interpreted the data, read the manuscript, and approved the final vision.

## Abbreviations

BMI: body mass index
CI: confidence interval
ER: Estrogen Receptor
FFQ: food frequency questionnaire
FIGO: International Federation of Gynecology and Obstetrics
HR: hazard ratio
Ref: reference
ROS: reactive oxygen species
IHC: immunohistochemistry
IQR: interquartile
MET: metabolic equivalents of task
OOPS: Ovarian Cancer Follow-Up Study
OS: overall survival
OC: ovarian cancer
PR: Progestogen Receptor
WT-1: Wilms’ tumor-1
SD: standard deviation.
