# Supplementary material for: Phytosterol intake and overall survival in newly diagnosed ovarian cancer patients: An ambispective cohort study
Source: Front Nutr. 2022 Aug 25;9:974367. doi: 10.3389/fnut.2022.974367 (PMC9452643; doi:10.3389/fnut.2022.974367)
Supplement: Supplementary file 1 [file Data_Sheet_1.docx]

**Supplementary Table 1.** **Selected clinical characteristics and associations with overall survival among ovarian cancer patients**

| **Characteristics** | **No. of deaths/total (%)** | **Adjusted HR ^*^ (95% CI)** |
| --- | --- | --- |
| **Age at diagnosis (years)** |  |  |
| ≤ 50 | 45/258 (17.44) | 1.00 (Ref) |
| > 50 | 85/445 (19.10) | 1.24 (0.85-1.79) |
| **Histological type** |  |  |
| Serous | 92/479 (19.21) | 1.00 (Ref) |
| Non-serous | 38/224 (16.96) | 1.71 (1.11-2.66) |
| **Histopathologic grade** |  |  |
| Well differentiated | 5/56 (8.93) | 1.00 (Ref) |
| Moderately differentiated | 7/48 (14.58) | 1.12 (0.35-3.57) |
| Poorly differentiated | 118/599 (19.70) | 1.76 (0.70-4.43) |
| **FIGO stage** |  |  |
| Ⅰ-Ⅱ | 41/342 (11.99) | 1.00 (Ref) |
| Ⅲ-Ⅳ | 89/338 (26.33) | 2.54 (1.65-3.91) |
| **Residual lesions** |  |  |
| No | 82/553 (14.83) | 1.00 (Ref) |
| < 1 cm | 31/106 (29.25) | 1.73 (1.11-2.68) |
| ≥ 1 cm | 17/44 (38.64) | 2.41 (1.39-4.16) |
| **Comorbidities** |  |  |
| No | 74/393 (18.83) | 1.00 (Ref) |
| Yes | 56/310 (18.06) | 0.97 (0.68-1.38) |

Abbreviations: CI, confidence interval; FIGO, Federation International of Gynecology and Obstetrics; HR, hazard ratio; Ref, reference.

**^*^** Mutually adjusted for all other variables listed in the table.

**Supplementary Table 2.** **Selected immunohistochemical biomarkers and associations with overall survival among ovarian cancer patients**

| **Characteristics** | **No. of deaths/total (%)** | **Adjusted HR ^*^ (95% CI)** |
| --- | --- | --- |
| **WT-1** |  |  |
| Positive | 59/378 (15.61) | 1.00 (Ref) |
| Negative | 44/190 (23.16) | 2.42 (1.51-3.87) |
| **ER** |  |  |
| Positive | 81/454 (17.84) | 1.00 (Ref) |
| Negative | 30/129 (23.26) | 2.09 (1.24-3.52) |
| **PR** |  |  |
| Positive | 50/321 (15.58) | 1.00 (Ref) |
| Negative | 61/262 (23.28) | 1.60 (1.07-2.38) |
| **Vimentin** |  |  |
| Positive | 29/156 (18.59) | 1.00 (Ref) |
| Negative | 65/359 (18.11) | 0.84 (0.51-1.38) |
| **P53** |  |  |
| Positive | 92/473 (19.45) | 1.00 (Ref) |
| Negative | 27/151 (17.88) | 0.94 (0.61-1.44) |

Abbreviations: CI, confidence interval; ER, Estrogen Receptor; HR, hazard ratio; PR, Progestogen Receptor; Ref, reference; WT-1, Wilms’ tumor-1.

**^*^** Adjusted for age at diagnosis (< 50 or ≥ 50 years), FIGO stage (I–II, III–IV, and unknown), histological type (serous or non-serous), histopathologic grade (well, moderate, and poorly differentiated), residual lesions (none, < 1, and ≥ 1 cm), and comorbidities (yes or no).

**Supplementary Table 3. Adjusted hazard ratio (HR) and 95% confidence interval (CI) of overall survival by the tertiles of phytosterol intake among 703 ovarian cancer patients: using nutrient-density method** ^*^

| **Variables** | **Tertiles of intake ^**^** | | | ***P* trend ^†^** |
| --- | --- | --- | --- | --- |
|  | **T1** | **T2** | **T3** |  |
| **Total phytosterols (****mg/1000kcal)** | 1.00 (Ref) | 0.54 (0.34-0.87) | 0.64 (0.39-1.05) | 0.11 |
| **Campestanol (mg/000kcal)** | 1.00 (Ref) | 0.79 (0.50-1.24) | 0.64 (0.34-1.24) | 0.20 |
| **β-Sitostanol (mg/1000kcal)** | 1.00 (Ref) | 0.65 (0.42-1.02) | 0.60 (0.35-1.03) | 0.07 |
| **Campesterol (mg/1000kcal)** | 1.00 (Ref) | 0.55 (0.35-0.88) | 0.50 (0.27-0.91) | < 0.05 |
| **Stigmasterol (mg/1000kcal)** | 1.00 (Ref) | 0.60 (0.38-0.95) | 0.63 (0.38-1.04) | 0.09 |
| **β-Sitosterol (mg/1000kcal)** | 1.00 (Ref) | 0.55 (0.34-0.89) | 0.67 (0.42-1.08) | 0.15 |

Abbreviations: CI, confidence interval; HR, hazard ratio; Ref, reference; T, tertile.

The analyses used three categories of total phytosterols (T1< 28.97, T2 28.97-42.58, and T3 ≥ 42.58mg/1000kcal), campestanol (T1: < 0.21, T2: 0.21-0.38, and T3: ≥ 0.38mg/1000kcal), β-sitostanol (T1:< 1.34, T2: 1.34-2.03, and T3: ≥ 2.03mg/1000kcal), campesterol (T1: < 3.36, T2: 3.36-5.18, and T3: ≥ 5.18mg/1000kcal), stigmasterol (T1: < 3.89, T2: 3.89-5.76, and T3: ≥ 5.76mg/1000kcal), and β-sitosterol (T1: < 20.35, T2: 20.35-29.89, and T3: ≥ 29.89mg/1000kcal).

**^*^** HR and 95% CI were calculated with the use of the Cox proportional hazards regression model.

**^**^** Energy-adjusted mean intake per day using the nutrient density method.

**^†^** Test for trend based on variables containing the median value for each tertile.

HR and 95% CI were calculated with the use of the Cox proportional hazards regression model with adjustment for age at diagnosis, education, cigarette smoking, alcohol drinking, monthly household income, dietary change, menopausal status, parity, body mass index, physical activity, FIGO stage, histological type, histopathologic grade, residual lesions, comorbidities, and total energy, isoflavone, and monounsaturated fatty acid intake.
